# Association between longer duration of citrate accumulation and 90-day mortality of acute-on-chronic liver failure

**DOI:** 10.1186/s13054-021-03819-8

**Published:** 2021-11-11

**Authors:** Ming Wang, Yuanji Ma, Lingyao Du, Hong Tang, Lang Bai

**Affiliations:** Center of Infectious Diseases, West China Hospital of Sichuan University, No. 37 GuoXue Xiang, Wuhou District, Chengdu, 610041 China

Regional citrate anticoagulation (RCA) is an optional anticoagulant for plasma adsorption (PA) plus plasma exchange (PE) therapy in patients with acute-on-chronic liver failure (ACLF), but with risk of transient citrate accumulation due to plasma and citrate [1]. Regardless of the anticoagulants: heparin or citrate, some patients would suffer from longer duration of citrate accumulation (LDCA), defined as the presence of citrate accumulation 2 h after PA plus PE therapy with RCA [1, 2]. However, whether citrate accumulation itself would lead to poor prognosis remains uncertain.

We conducted a retrospective study based on medical records to assess the association between LDCA and prognosis of hepatitis B virus (HBV)-related ACLF. Methods and some data from this cohort have been published already [2]. We kept to follow-up these patients for another 90 days after acquiring further ethical approval and registered this study with ChiCTR-OON-17013631. HBV-ACLF was diagnosed according to COSSH ACLF criteria [3]. Citrate accumulation was defined as the ratio of total calcium (Ca_tot_) to ionized calcium (Ca_ion_), (Ca_tot_/Ca_ion_), over or equal to 2.5 (Ca_tot_/Ca_ion_ ≥ 2.5) [1, 2]. Cox proportional hazards models were applied to evaluate the association of LDCA with outcome.

From January 2018 to December 2019, we reviewed the data of 258 patients who fulfilled the HBV-ACLF criteria and received PA plus PE therapy with RCA. LDCA patients (*N* = 76) were more often female and older and had worse severity of disease condition than non-LDCA patients (*N* = 182) (Table 1). There was no significant difference in indicators, such as intracorporeal and extracorporeal Ca_tot_ and Ca_ion_, representing patients receiving similar RCA during and after the first session of PA plus PE therapy with RCA.

**Table 1:** Characteristics of ACLF patients with or without LDCA

	Patients with LDCA (*N* = 76)	Patients without LDCA (*N* = 182)	*p*
Female	25 (32.9%)	12 (6.6%)	< 0.001
Age(years)	52.2 ± 10.9	43.8 ± 11.2	< 0.001
Liver cirrhosis	61 (80.3%)	141 (77.5%)	0.620
Causes of liver disease			0.963
HBV infection only	57 (75.0%)	137 (75.3%)	
HBV infection plus other causes	19 (25.0%)	45 (24.7%)	
Comorbidities			0.112
No	59 (77.6%)	156 (85.7%)	
Yes	17 (22.4%)	26 (14.3%)	
Disease severity assessment			
COSSHACLF score	7.1 ± 1.0	6.3 ± 0.8	< 0.001
CLIF-C ACLF score	38.9 ± 6.9	32.7 ± 6.5	< 0.001
AARCACLF score	10.7 ± 1.6	9.6 ± 1.5	< 0.001
MELD score	29.8 ± 5.5	25.7 ± 3.9	< 0.001
*Laboratory examination*			
PT-INR	2.36 (1.95–2.81)	2.06 (1.75–2.44)	0.009
Serum creatinine (× ULN)	0.97 (0.80–1.32)	0.80 (0.65–0.88)	< 0.001
Total bilirubin (μmol/L)	431.0 ± 135.4	421.9 ± 120.0	0.495
Direct bilirubin to total bilirubin ratio	0.75 (0.70–0.82)	0.80 (0.73–0.86)	0.009
Alanine aminotransferase (IU/L)	140 (56–300)	124 (66–245)	0.891
Aspartate aminotransferase (IU/L)	139 (76–227)	116 (88–192)	0.133
Aspartate aminotransferase to alanine aminotransferase ratio	1.13 (0.65–1.92)	1.06 (0.64–1.53)	0.495
Albumin (g/L)	31.8 ± 3.6	31.8 ± 4.0	0.742
Albumin to globulin ratio	1.2 ± 0.4	1.2 ± 0.4	0.041
Ammonia (mmol/L)	77.6 (58.0–117.8)	79.1 (60.9–110.2)	0.891
Lactate (mmol/L)	2.98 (2.03–3.89)	2.40 (1.90–3.00)	< 0.001
Serum sodium (mmol/L)	130.7 ± 15.8	134.5 ± 4.1	0.009
Serum potassium (mmol/L)	3.44 ± 0.55	3.46 ± 0.58	0.866
Serum chloride (mmol/L)	93.9 ± 5.6	97.3 ± 4.4	< 0.001
Hemoglobin (g/L)	111 ± 18	122 ± 20	0.002
Platelets (× 10^9^/L)	83 (48–114)	91 (64–124)	0.180
White blood cells (× 10^9^/L)	7.87 ± 4.08	7.47 ± 3.48	0.495
Intracorporeal Ca_tot_ before PA therapy (mmol/L)	2.16 ± 0.15	2.13 ± 0.13	0.133
Intracorporeal Ca_ion_ before PA therapy (mmol/L)	1.020 ± 0.089	1.051 ± 0.076	0.123
Intracorporeal Ca_tot_ during PA therapy (mmol/L)	2.06 ± 0.21	1.97 ± 0.24	0.595
Intracorporeal Ca_ion_ during PA therapy (mmol/L)	0.749 ± 0.098	0.808 ± 0.109	0.262
Extracorporeal Ca_ion_ during PA therapy (mmol/L)	0.167 (0.132–0.233)	0.184 (0.145–0.238)	0.345
Intracorporeal Ca_tot_ 2 h after PE therapy (mmol/L)	2.65 ± 0.26	2.46 ± 0.18	< 0.001
Intracorporeal Ca_ion_ 2 h after PE therapy (mmol/L)	0.962 ± 0.100	1.103 ± 0.081	< 0.001
Ca_tot_/Ca_ion_ 2 h after PE therapy	2.70 (2.58–2.90)	2.22 (2.14–2.32)	< 0.001
Anion gap 2 h after PE therapy (mmol/L)	7.67 ± 2.90	6.85 ± 2.34	0.010
DPMAS plus PE therapy with RCA			
Sessions	3.0 (2.3–5.0)	4.0 (3.0–6.0)	0.204
Days from the first to the last sessions	7.0 (4.0–14.0)	8.0 (5.0–14.0)	0.292
90-day prognosis (death)	48 (63.2%)	59 (32.4%)	< 0.001

Quantitative data are represented as mean ± SD (normally distributed data) or median (interquartile range) (non-normally distributed data) and compared by Mood's median test. Qualitative data are represented as frequencies (proportion) and compared by Chi-squared test

ACLF, Acute-on-chronic liver failure; LDCA, longer duration of citrate accumulation; HBV, hepatitis B virus; COSSH, Chinese Group on the Study of Severe Hepatitis B; CLIF-C, European Association for the Study of the Liver—Chronic Liver Failure-Consortium; AARC, APASL ACLF Research Consortium; APASL, Asian Pacific Association for the Study of the Liver; MELD, Model for End-Stage Liver Disease; PT-INR, international normalized ratio (INR) of prothrombin time (PT); ULN, upper limit of normal; PA, plasma adsorption; PE, plasma exchange; Ca_tot_, total calcium; Ca_ion_, ionized calcium; Ca_tot_/Ca_ion_, Ca_tot_ to Ca_ion_ ratio

The 90-day mortality of LDCA patients was much higher than that of non-LDCA patients (63.2% vs. 32.4%, log-rank *p* < 0.001). Compared with non-LDCA patients, LDCA patients had much higher 90-day mortality risk (crude hazard ratio (HR) (95% confidence interval (CI)), 2.62 (1.79–3.84)) (Table 2). However, no significant differences in 90-day mortality risk were observed with the Cox proportional hazards models established with LDCA, age, gender, liver cirrhosis, HBV DNA, other co-existing liver diseases, comorbidities, and disease severity (Model 1, COSSH ACLF score; Model 2, CLIF-C ACLF score; Model 3, AARC ACLF score; Model 4, MELD score): Model 1 adjusted HR (95% CI), 1.07 (0.66–1.73); Model 2, 1.49 (0.95–2.36); Model 3, 1.41 (0.90–2.22); Model 4, 1.05 (0.65–1.72) (Table 2). Similarly, no significant differences in 90-day mortality risk were observed with similar Cox models established with citrate level indicators (Model 5, Ca_tot_/Ca_ion_ ≥ 2.25; Model 6, Ca_tot_/Ca_ion_; Model 7, anion gap), disease severity (COSSH ACLF score), and the others mentioned above: Model 5, 1.28 (0.78–2.08); Model 6, 1.56 (0.74–3.27); Model 7, 1.06 (0.97–1.16). The disease severity was the independent risk factor of 90-day mortality (Model 1–7, all adjusted HR > 1, all *p* < 0.001).

**Table 2:** LDCA and other factors associated with risk of 90-day mortality in ACLF patients

	Crude HR (95% CI)	Adjusted HR^▲^ (95% CI)			
		Model 1	Model 2	Model 3	Model 4
LDCA					
No	1	1	1	1	1
Yes	2.62 (1.79–3.84)***	1.07 (0.66–1.73)	1.49 (0.95–2.36)	1.41 (0.90–2.22)	1.05 (0.65–1.72)
Age (years)	1.03 (1.01–1.05)***	0.99 (0.97–1.02)	0.97 (0.94–0.99)**	1.02 (1.00–1.04)	1.01 (0.99–1.03)
Gender					
Male	1	1	1	1	1
Female	1.84 (1.15–2.94)*	1.24 (0.73–2.08)	1.04 (0.62–1.76)	1.25 (0.74–2.09)	1.81 (1.07–3.08)*
Liver cirrhosis					
No	1	1	1	1	1
Yes	2.51 (1.37–4.57)**	1.66 (0.90–3.08)	2.14 (1.17–3.95)*	2.20 (1.19–4.06)*	1.97 (1.07–3.65)*
HBV DNA (log10 IU/mL)	0.98 (0.89–1.09)	1.02 (0.92–1.13)	1.00 (0.90–1.12)	1.00 (0.90–1.12)	1.01 (0.90–1.13)
Etiology					
HBV infection only	1	1	1	1	1
HBV infection plus other causes^■^	0.93 (0.60–1.45)	1.07 (0.68–1.69)	1.07 (0.68–1.68)	1.06 (0.67–1.67)	0.82 (0.51–1.29)
Comorbidity^◆^					
No	1	1	1	1	1
Yes	1.86 (1.20–2.90)**	1.74 (1.05–2.87)*	1.56 (0.96–2.55)	1.60 (0.98–2.61)	1.75 (1.06–2.90)*
Disease severity					
COSSH ACLFscore	2.78 (2.31–3.34)***	2.72 (2.17–3.40)***	–	–	–
CLIF-C ACLF score	1.13 (1.09–1.16)***	–	1.15 (1.10–1.19)***	–	–
AARCACLF score	1.60 (1.41–1.82)***	–	–	1.59 (1.38–1.83)***	–
MELD score	1.16 (1.12–1.20)***	–	–	–	1.17 (1.12–1.22)***

HBV infection plus other causes^■^: the ones having HBV infection plus any one of other co-existing liver diseases was classified to this subgroup

Comorbidity^◆^: the ones having any one of comorbidities were classified as the comorbidity group

Adjusted HR^▲^: multivariable Cox regression analysis includes LDCA (yes vs no), age (continuous years), gender (female vs male), liver cirrhosis (yes vs no), HBV DNA (continuouslog10 IU/mL), other co-existing liver diseases (viral infections other than hepatitis B virus, alcoholic liver disease, non-alcoholic fatty liver, immune related liver disease, drug induced liver injury, and other liver diseases), comorbidities (chronic obstructive pulmonary disease, diabetes mellitus, coronary heart disease, primary hypertension, chronic kidney disease, and other chronic diseases), and disease severity (model 1, COSSH ACLF score; model 2, CLIF-C ACLF score; model 3, AARC ACLF score; model 4, MELD score)

ACLF, Acute-on-chronic liver failure; LDCA, longer duration of citrate accumulation; HR, hazard ratio; CI, confidence interval; COSSH, Chinese Group on the Study of Severe Hepatitis B; CLIF-C, European Association for the Study of the Liver—Chronic Liver Failure-Consortium; AARC, APASL ACLF Research Consortium; APASL, Asian Pacific Association for the Study of the Liver; MELD, Model for End-Stage Liver Disease

***p < 0.001; **p < 0.01; *p < 0.05

Our study proved that ACLF patients with LDCA would suffer higher 90-day mortality. This finding was in accordance with the results in critically ill patients undergoing continuous renal replacement therapy with RCA [4]. However, no significant differences in 90-day mortality risk were found in ACLF patients with or without LDCA. As RCA brings no alteration of pro- and anti-coagulation function and ACLF patients have re-balanced but fragile coagulation function [1, 5], our new results would support the use of RCA with caution in ACLF patients. Adequate training, experienced operation, and well-developed safety protocols would further expand indications of RCA [6].

Our study for the first time assessed the association between LDCA and prognosis in ACLF patients treated with PA plus PE therapy with RCA. There were limitations: monocentric retrospective design, only HBV-ALCF cases, and applying Ca_tot_/Ca_ion_ instead of directly measuring plasma citrate concentration to reflect citrate accumulation.

## Data Availability

The datasets used and/or analyzed during the current study are available from the corresponding author on reasonable request.

## Abbreviations

AARC: Asian Pacific Association for the Study of the Liver—ACLF Research Consortium
ACLF: Acute-on-chronic liver failure
Ca_tot_: Total calcium
Ca_ion_: Ionized calcium
CI: Confidence interval
CLIF-C: European Association for the Study of the Liver—Chronic Liver Failure-Consortium
COSSH: Chinese Group on the Study of Severe Hepatitis B
HBV: Hepatitis B virus
HR: Hazard ratio
LDCA: Longer duration of citrate accumulation
MELD: Model for end-stage liver disease
PA: Plasma adsorption
PE: Plasma exchange
RCA: Regional citrate anticoagulation
